# Cardiorenal Syndrome: Emerging Role of Medical Imaging for Clinical Diagnosis and Management

**DOI:** 10.3390/jpm11080734

**Published:** 2021-07-28

**Authors:** Ling Lin, Xuhui Zhou, Ilona A. Dekkers, Hildo J. Lamb

**Affiliations:** 1 Cardiovascular Imaging Group (CVIG), Department of Radiology, Leiden University Medical Center, 2333 ZA Leiden, The Netherlands; l.lin@lumc.nl (L.L.); i.a.dekkers@lumc.nl (I.A.D.); h.j.lamb@lumc.nl (H.J.L.); 2 Department of Radiology, The Eighth Affiliated Hospital of Sun Yat-sen University, Shenzhen 510833, China

**Keywords:** cardiorenal syndrome, imaging biomarker, tissue characterization

## Abstract

Cardiorenal syndrome (CRS) concerns the interconnection between heart and kidneys in which the dysfunction of one organ leads to abnormalities of the other. The main clinical challenges associated with cardiorenal syndrome are the lack of tools for early diagnosis, prognosis, and evaluation of therapeutic effects. Ultrasound, computed tomography, nuclear medicine, and magnetic resonance imaging are increasingly used for clinical management of cardiovascular and renal diseases. In the last decade, rapid development of imaging techniques provides a number of promising biomarkers for functional evaluation and tissue characterization. This review summarizes the applicability as well as the future technological potential of each imaging modality in the assessment of CRS. Furthermore, opportunities for a comprehensive imaging approach for the evaluation of CRS are defined.

## 1. Introduction

Cardiorenal syndrome (CRS) is an umbrella term describing the interactions between concomitant cardiac and renal dysfunctions, in which acute or chronic dysfunction of one organ may induce or precipitate dysfunction of the other [1]. CRS has been associated with increased morbidity and poor clinical outcomes, leading to high economic and societal burden [2]. The estimated incidence of acute kidney injury is 24–45% in acute decompensated heart failure and 9–19% in acute coronary syndrome [3]. The prevalence of impaired renal function is high in chronic cardiovascular diseases, and around 40–60% in chronic heart failure [4]. The combination of renal dysfunction with chronic heart failure is predictive of adverse clinical outcomes [5]. Nearly 50% of deaths in all age groups of patients with chronic kidney disease (CKD) can be attributed to cardiovascular causes [6]. CRS is also frequently observed in acute or chronic systemic conditions, such as sepsis and diabetes mellitus, and is associated with worse outcomes [7].

**Figure 1:**
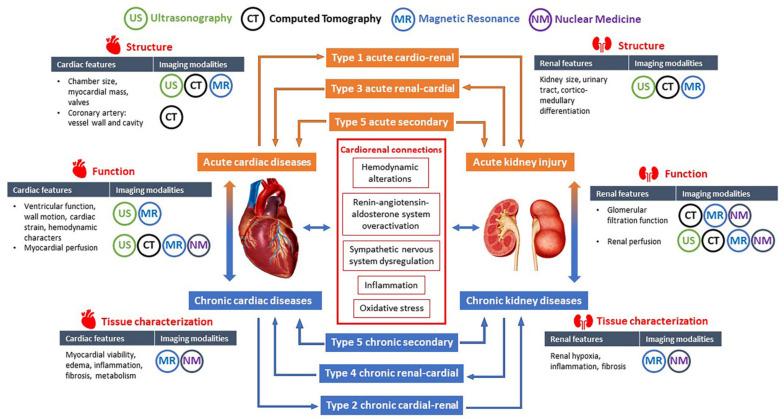
Overview of the contents. The heart and kidneys interact through multiple pathophysiological pathways which may lead to five subtypes of CRS. The structural, functional and tissue texture changes in the heart and kidneys can be evaluated using different imaging modalities including ultrasonography, computed tomography, magnetic resonance, and nuclear medicine.

Despite the existing literature on the classification and management of CRS, the clinical diagnosis and treatment evaluation remains difficult due to the lack of clinical practice guidelines [8]. This has led to increased research interests, including studies focused on the early diagnosis and clinical management of CRS. The potential value of imaging biomarkers for the early detection of cardiac abnormalities in CRS has been underlined in the scientific statement from the American Heart Association [8]. Ultrasonography is currently the first-line imaging modality for structural and functional assessment of the heart, and structural assessment of the kidneys. Computed tomography (CT), nuclear imaging, and magnetic resonance imaging (MRI) have been widely used for various purposes in clinical management of cardiovascular diseases and kidney diseases. Recent technological advancements in medical imaging provides a number of promising biomarkers for the diagnosis and prognosis of CRS, and opportunities for personalized medicine. In this review, we will summarize the cardiovascular and renal imaging techniques related to CRS and the potential utility of these techniques for the diagnosis and follow-up of acute and chronic CRS (Figure 1). Finally, comprehensive imaging protocols that can be incorporated into future research studies and clinical trials will be proposed.

## 2. Classification, Pathophysiology, and Clinical Management of CRS

### 2.1. Classification of CRS

Cardio-renal syndrome can be classified into five subtypes [1], with type 1 and 2 describing renal dysfunction sequent to initial acute and chronic cardiac insults, type 3 and 4 describing renocardiac syndrome after the initial insult of kidney disease, and type 5 representing secondary CRS in systemic diseases (Table 1). Although this classification simplifies the clinical concept of CRS, overlap between different subtypes and progression from one subtype to another has frequently been observed [9]. For example, it is challenging to differentiate type 2 CRS from type 4 CRS as chronic heart diseases and chronic kidney diseases frequently co-exist [10,11]. Moreover, the development of CRS is often complicated by several interconnected conditions, such as diabetes, hypertension, atherosclerosis, endothelial cell dysfunction, chronic inflammation, and anemia, rendering difficulties in defining the temporal progression patterns of CRS [8]. An alternative classification of CRS was proposed by Hatamizadeh et al. based on clinical manifestations rather than the organ that initiated the process [12], but has not received wide acceptance.

### 2.2. Pathophysiology of CRS

The exact pathophysiological mechanisms of each type of CRS have not been fully elucidated. Previously, decreased cardiac output and arterial underfilling induced neurohumoral activations were believed to be the sole pathogenesis of CRS [13]. However, studies in the past decades demonstrated that decreased arterial flow does not fully explain the worsening renal function in CRS (60–63). Elevated central venous pressure has closer association with the reduction of renal perfusion than decreased cardiac output (61). Moreover, increasing evidence indicates that multiple pathophysiological processes contribute to the evolution of CRS [14]. Hemodynamic alterations, renin–angiotensin–aldosterone system (RAAS), sympathetic nervous system, inflammatory, and oxidative stress are considered as key connectors between heart and kidneys [15,16]. Other contributing factors such as biochemical perturbations, immune responses, atherosclerosis and anemia–inflammation–bone mineral axis, can also accelerate the development of CRS, especially in chronic heart failure and CKD [8,15,17]. These pathways are interconnected and exhibit varied clinical importance across different subtypes of CRS [3,18]. 

Hemodynamic alterations, especially right-sided heart dysfunction, is believed to be of critical importance in the development of acute CRS (type 1 and type 3) [19]. In type 1 CRS, increased central venous pressure results in renal venous congestion, which may lead to impaired glomerular filtration, tissue hypoxia and renal fibrogenesis. These pathological changes induce or aggravate renal dysfunction, which in return exacerbates fluid overload leading to further deterioration of cardiac function [4,15]. In type 3 CRS, acute heart injury can be caused by excessive cytokines due to AKI, and by indirect mechanisms including neurohumoral activation, electrolytes disturbances, uremia, and acidemia [20,21]. 

Non-hemodynamic pathways play a more critical role in chronic CRS (type 2 and type 4). Activation of RAAS and stimulation of sympathetic nervous system are features of both heart failure and CKD. Persistent activation of RAAS leads to peripheral vasoconstriction, exacerbated fluid overload, and sympathetic nervous system overactivation [16,22]. Sympathetic overactivation in return can stimulate RAAS via renin release, resulting in a vicious circle [16]. Chronically increased release of aldosterone is the major deleterious component of RAAS and has been associated with both myocardial and renal interstitial fibrosis [23]. Increased oxidative stress due to chronic RAAS activation has also been associated with renal injury and fluid retention [24]. Inflammation cascade can be triggered by and potentiate the other cardiorenal connectors, including the overactivation of RAAS and sympathetic nervous system, and increased oxidative stress. Systemic inflammation is associated with myocardial and renal dysfunction and interstitial fibrosis [19,25]. 

Fibrosis has been considered as a key driver in the pathophysiology of chronic CRS [18]. Fibrogenic responses have short-term adaptive features in the early phases of cardiac and renal diseases. However, when it progresses chronically, fibrosis can lead to myocardial and renal parenchymal scarring, cellular dysfunction and ultimately organ failure [26]. Fibrosis of heart and kidneys has also been found in a number of CRS risk factors, including aging, hypertension, diabetes mellitus, and obesity [27]. Based on these findings, a new pragmatic and dynamic cardiorenal integrative concept of CRS has been proposed, in which patients may be categorized according to the predominant pathophysiological mechanism, rather than clinical presentation [18]. This strategy has the potential to facilitate clinical interventions for CRS in the future. 

### 2.3. Current Difficulties in Diagnosis and Management of CRS

In most circumstances, the complex interconnected pathways between heart and kidneys have already been activated by the time clinical manifestations are detectable. Both heart and kidneys have substantial functional reserve, which makes it difficult to prevent or reverse the adverse impacts of CRS at an early phase. While all types of CRSs are faced with difficulties in early diagnosis and prognosis, the dominant clinical challenges distinguish between acute CRS and chronic CRS (Table 2).

The main challenges in acute CRS are related to AKI (Table 2). Currently, AKI is diagnosed based on serum creatinine (SCr) level and oliguria [28]. However, SCr cannot detect early kidney dysfunction, since it remains within normal range before half the kidney function is lost, resulting in a lag between kidney insult and the elevation of SCr [29]. On the other hand, pseudo-worsening of kidney function may occur due to hemodynamic changes in patients with heart failure, which is difficult to be differentiated from true kidney injury [30]. Apart from the inability to prevent or early identify AKI, the lack of sensitive tools to track the progression from AKI to CKD also challenges the clinical management of AKI. It has been reported that AKI is independently associated with higher rates of incident CKD [31]. Moreover, kidney dysfunction may decrease the efficiency of diuretics in patients with heart failure, resulting in diuretic resistance and worsening of congestion, which in return deteriorates the heart and kidney functions [19]. Strategies to prevent AKI or early interventions in the course of AKI remain to be investigated to reduce the risk of future adverse renal and cardiac outcomes. In addition, there is a demanding need of guidance on cardiac- and reno-protective therapies in acute CRS. 

In chronic CRS, however, the main difficulties lie in the cardiac aspect (Table 2). Patients with CKD suffer from a high risk of cardiovascular diseases that is disproportionate to the risk expected in general population [32]. In early-stage CKD, the risk of cardiovascular death far exceeds the risk of progressing to dialysis [33]. Previous studies suggested that subtle alterations in cardiac structure and function could occur very early in the progression of CKD, even when SCr is still within the normal range [34]. In addition, nonatheromatous processes appear to predominate the progression of cardiovascular disease in CKD, which could explain the lower effect of standard treatment on decreasing cardiovascular mortality in CKD patients than in general population [35]. Early detection of cardiovascular abnormality in CKD is challenging due to lack of overt symptoms and preserved left ventricular systolic function [36]. 

Despite the amount of effort in research studies of novel serum and urinary biomarkers, it remains unclear whether and to what extent these biomarkers can be involved in clinical management of CRS [37]. Moreover, the global availability of biomarker technology is another obstacle upon implementing this strategy in clinical practice. Imaging techniques that provide quantitative information on blood flow, perfusion, diffusion, tissue oxygenation, and interstitial fibrosis without radiation or potential risks of contrast agents offer possibilities of noninvasive assessment of preclinical pathophysiological changes in the heart and kidneys at the early phase of CRS.

## 3. Cardiovascular and Renal Imaging Techniques Related to CRS

Different imaging modalities can be applied in relation to CRS that enabling comprehensive assessment of both morphology and function (Table 3). Although further validations are needed for some of these techniques, a number of promising imaging biomarkers that might be valuable for the clinical management of CRS are discussed below. 

### 3.1. Cardiovascular Imaging Techniques

#### 3.1.1. Transthoracic Echocardiography

Transthoracic echocardiography (TTE) is the most available non-invasive imaging technique to measure the dimensions of cardiac chambers and to estimate ventricular functions. TTE-measured left ventricular ejection fraction is the first-line tool to differentiate between heart failure with reduced ejection fraction and heart failure with reserved ejection fraction [38]. TTE can rapidly identify wall motion abnormalities, valvular diseases and pericardial effusion. Various hemodynamic markers can be estimated by Doppler imaging, such as mitral inflow and mitral annulus motion, left atrial volume and pressure, left ventricular filling pressure, systolic pulmonary artery pressure, pulmonary capillary wedge pressure, and right ventricular function [39]. Myocardial strain based on speckle tracking technique can be used to quantify ventricular wall deformation, with global longitudinal strain being more sensitive to subtle impairment of ventricular systolic function than ejection fraction [40]. Fast and cost-effective as it is, TTE-derived imaging biomarkers can be limited by inadequate acoustic window, poor Doppler signals and operator-dependent variations. The utility of ultrasonic enhancing agent improves structural and functional evaluations of various cardiovascular diseases [41]. Enhanced TTE also enables the assessment of myocardial perfusion at rest or with vasodilator-induced stress [41].

#### 3.1.2. Cardiovascular Magnetic Resonance 

**Figure 2:**
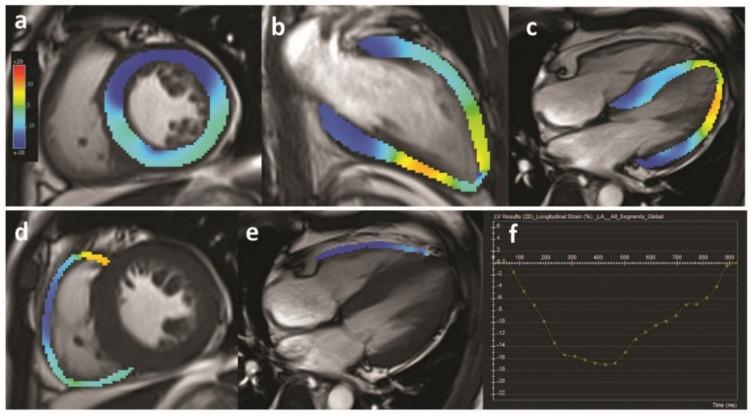
Example of myocardial strain analysis using MRI in a patient with CKD. Quantification of left ventricular strain (**a**–**c**) and right ventricular strain (**d**,**e**) parameter is visualized by colored overlay on cine images. (**f**) is an example of strain–time curve of the left ventricular global longitudinal strain within one cardiac cycle.

Over the last decade, cardiovascular magnetic resonance (CMR) has gained increasing acknowledgement in the clinical management of cardiovascular diseases [42,43]. CMR-measured biventricular volumes, systolic function and myocardial mass are gold-standard imaging biomarkers [44], particularly right ventricular geometry and function. Myocardial strain parameters can also be generated from CMR using feature/tissue tracking post-processing algorithms, free from the suboptimal acoustic window and dropouts in TTE [45] (Figure 2). Velocity encoding using phase contrast technique enables quantitative evaluation of valvular diseases and shunt evaluation by CMR. Using gadolinium-based contrast agents, myocardial perfusion and myocardial fibrosis or infiltration can be assessed and quantified. Late gadolinium enhancement is the best non-invasive technique to visualize focal fibrosis [46]. Extracellular volume fraction (ECV) calculated by pre- and post-contrast T1 relaxation time is useful for detecting diffused myocardial fibrosis [47]. However, the application of contrast-enhanced CMR in CRS is limited in patients with severely decreased renal function (eGFR < 30 mL/min/1.73 m^2^), considering the potential increased risk of gadolinium retention and nephrogenic systemic fibrosis in patients with renal dysfunction [48,49], but these risks are less clear for the more modern macrocyclic contrast agents [48]. 

**Figure 3:**
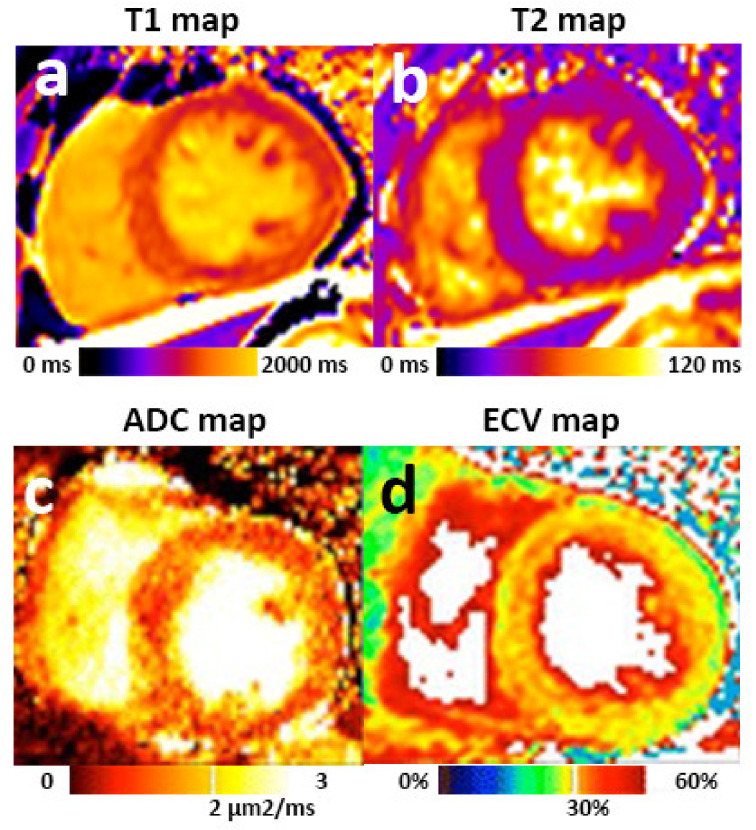
Myocardial tissue characterization by multiparametric MRI. Mid-cavity short-axis T1 map (**a**) and T2 map (**b**) of a patient with CKD. Myocardial T1 and T2 values can be quantified and compared with local references. ADC (**c**) and ECV (**d**) images demonstrated diffused “pepper like” hyper intensity texture in a patient with hypertrophic cardiomyopathy. Images (**c**,**d**) were adapted from published article [52] under a Creative Commons license.

Non-contrast tissue characterization techniques including T1 mapping, T2 mapping and diffusion weighted imaging (DWI) provide unique opportunities to identify microstructural changes in myocardium (Figure 3). T1 and T2 mapping are increasingly used in clinical settings. T1 mapping quantifies the longitudinal and T2 mapping transverse magnetization relaxation times of the hydrogen nucleus proton per voxel, which can reflect the presence of fibrosis, fat, edema, and iron deposition [50]. Myocardial T1 and T2 values have been applied to detect abnormalities in myocardial tissue composition in various diseases that related to CRS—including heart failure, ischemic heart diseases, hypertensive cardiomyopathy, diabetic cardiomyopathy, and uremic cardiomyopathy [50,51]. DWI characterizes the motion of water molecules in microstructural changes, and quantifies it as apparent diffusion coefficient (ADC). Previous studies suggested that DWI was able to detect and quantify the degree of myocardial fibrosis, with the minimum amount of fibrosis larger than 20% [52,53,54].

#### 3.1.3. Cardiac Computed Tomography 

Computed tomography (CT) coronary angiography is the most widely used noninvasive imaging technique for anatomical assessment of coronary artery disease (CAD). CT angiography with additional perfusion imaging allows for characterization of atherosclerosis in relation to myocardial ischemia, which has great potential clinical value [55]. CT-based fractional flow reserve allows for the quantification of the impaired maximal coronary flow induced by a stenosis, which is adapted from invasive coronary pressure measurement [56]. CT can also be used to estimate myocardial ECV, and is an attractive alternative to CMR to evaluate diffused myocardial fibrosis [57]. However, major challenges of CT are the limited temporal resolution, presence of beam and scatter artefacts, radiation dose, and low contrast-to-noise ratios [58,59,60]. Moreover, these CT techniques rely on iodinated contrast agents, which is associated with the risk of post-contrast AKI in patients with impaired renal function [61]. Without contrast agent, CT can be used to calculate coronary artery calcium score, which is a prognostic biomarker for CAD. 

#### 3.1.4. Nuclear Cardiac Imaging 

Nuclear cardiac imaging has played an important role in evaluating myocardial perfusion in ischemic heart diseases. Single-photon emission computed tomography (SPECT) is commonly employed for the diagnosis of CAD in patients with CKD [62]. However, SPECT only provides semi-quantitative assessment of myocardial perfusion, and has a wide range of sensitivity, specificity, and accuracy [63]. Quantitative positron emission tomography (PET), on the other hand, measures absolute myocardial blood flow and has shown greater prognostic value than SPECT in evaluation of patients with known or suspected CAD [64]. Currently four different tracers are used for clinical assessment of myocardial blood flow, which are ^82^Rb, ^13^N-ammonia, ^15^O-water, and ^18^F-flurpiridaz. ^15^O-water-PET is considered the clinical reference standard for non-invasive quantification of myocardial perfusion; however, important challenges include high-cost, limited visual assessment, and the lower spatial resolution of PET compared with CT or MRI perfusion imaging [65]. Myocardial metabolism alterations such as increased glucose utility and fatty acid oxidation can also be evaluated by ^18^F-fluoro-2-deoxyglucose [^18^F-FDG] PET and β-Methyl-p-[123I]-iodophenyl-pentadecanoic acid SPECT [63] Hybrid imaging such as SPECT-CT, PET-CT, and PET-MRI can generate multiple imaging biomarkers by single examination.

### 3.2. Renal Imaging Techniques

#### 3.2.1. Renal Ultrasonography

Renal ultrasonography is routinely used to assess renal morphology such as renal length, corticomedullary differentiation, and to identify obstruction. The usefulness of ultrasonography to identify the underlying cause of renal diseases is limited, furthermore no distinction between inflammation and fibrosis can be identified by echogenicity [66]. Renal Doppler sonography enables the quantification of renal blood flow and intrarenal hemodynamic changes, which are suggestive of renal dysfunction and/or microstructural alterations. Elevated values of renal resistive index are associated with poorer prognosis in various renal disorders and renal transplant [67]. Renal venous flow is one of the biomarkers for right-sided congestion, which is fundamental to the management of CRS. Contrast-enhanced ultrasonography has showed the ability to quantify regional renal perfusion and microvascular function in rat models, and is potentially feasible for early detection and monitoring of AKI [68,69].

#### 3.2.2. Renal Magnetic Resonance Imaging

**Figure 4:**
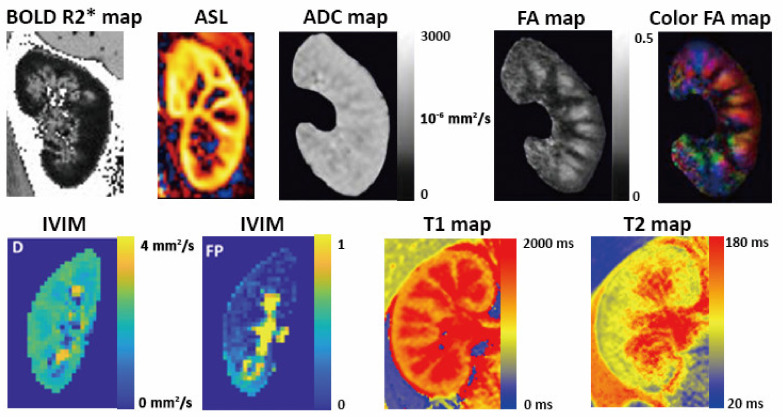
Multiparametric kidney MRI in healthy volunteers. BOLD R2* map is used to evaluate parenchyma oxygenation. Renal blood flow can be quantified from ASL perfusion weighted image. ADC and FA maps generated from DWI and DTI can be used to assess renal fibrosis. IVIM imaging evaluates true parenchyma diffusion by separate modeling. Renal T1 maps showing clear cortico-medullary differentiation in a healthy volunteer and T2 mapping are promising techniques to evaluate renal microstructure. The BOLD R2* map, ADC map, FA maps and IVIM images were adapted from the articles of Bane et al. [71], Adams et al. [76], and de Boer et al. [77] under Creative Commons licenses.

Initial applications of renal MRI have been focused on the visualization of renal and urogenital anatomy. Conventional renal MRI sequences can be used to measure total kidney volume, which is an FDA-approved prognostic biomarker [70], with higher accuracy compared with sonography. Recent research interest has been focused on the application of sequences that provide functional (BOLD, ASL) and microstructural (DWI, DTI, T1 mapping, T2 mapping) information without the need for gadolinium-based contrast agents [71,72,73,74,75] (Figure 4). 

Renal parenchymal oxygenation is of paramount importance in the pathophysiology of AKI and CKD [78]. Blood oxygen level dependent (BOLD) imaging can demonstrate tissue oxygen level using multi-echo T2*-weighted sequence based on the paramagnetic properties of deoxyhemoglobin. The strong correlation between renal T2* (R2*) and the invasive gold-standard tissue oxygen partial pressure has been validated in rat model [79]. The outer layer of medulla has higher sensitivity to hypoxia than the cortex, which is the physiological basis of the susceptibility to hypoxia injury. 

Arterial spin labeling (ASL) assesses tissue perfusion by labeling the water protons in the blood before they enter the tissue of interest, and subtracting the labeled image from a control image without labeling blood water. The signal intensity of the subtracted perfusion-weighted image is proportionate to perfusion. ASL has been widely used to calculate cerebral perfusion in various brain diseases [80]. Renal perfusion quantified by ASL has been validated by comparison with para-aminohippuric-acid clearance, which is the gold standard measurement of renal plasma flow, and with renal scintigraphy, demonstrating reproducible perfusion measurements [81,82]. High interstudy and interrater reproducibility of ASL in the quantification of cortical and medullary renal perfusion has been shown in healthy volunteers [83].

Renal DWI, diffusion tensor imaging (DTI), T1 and T2 mapping have been studied to assess interstitial fibrosis [84]. Renal cortex has higher ADC than medulla in healthy kidneys. As ADC is largely influenced by tubular flow and capillary perfusion, intravoxel incoherent motion (IVIM) is used to measure the true diffusion, alongside the pseudo-diffusion and flow fraction. DTI is a variation to DWI which measures the fractional anisotropy (FA); that is, the percentage of a tissue that displays oriented diffusion axes. Increased ADC and decreased FA can be biomarkers of fibrosis in CKD. Recent studies suggest that renal T1 mapping technique can be used to assess tissue changes in AKI and renal fibrosis in CKD in rat modal [85,86,87] as well as in human [88], with good reproducibility. 

#### 3.2.3. CT and Nuclear Medicine for Renal Imaging

CT and nuclear imaging are the most frequently used modalities after ultrasonography to assess renal morphology and function in clinical settings. However, the utility of renal CT in clinical management of CRS is limited due to radiation and the risk of post-contrast acute kidney injury in patients with impaired renal function (eGFR < 30 mL/min/1.73 m^2^). Dual-energy CT might offer opportunities to assess renal parenchyma without contrast agent. Renal nuclear imaging such as renal scintigraphy, SPECT, and PET have been used for quantification of GFR and renal perfusion. However, they are not ideal for frequent assessments due to radiation, thus not suitable for longitudinal surveillance of CRS.

## 4. Application of Imaging Biomarkers in Acute CRS

### 4.1. Echocardiographic and CMR Biomarkers for Diagnosis and Prognosis

Echocardiography not only is essential for diagnosing cardiovascular dysfunction in acute CRS, but also provides prognostic biomarkers. In a retrospective study of 30,681 patients, at least one type of CRS was detected in 8% patients, in whom decreased left ventricular ejection fraction, increased pulmonary artery pressure and larger right ventricular diameter derived by TTE were independent risk factors of the development of CRS [9]. This study also found that acute CRS is associated with the worst prognosis in comparison with chronic CRS and no CRS [9]. In a study of 1879 critical ill patients, right ventricular dysfunction assessed by TTE was an important determinant of AKI and AKI-related mortality [89]. 

CMR has been increasingly used in acute cardiovascular diseases such as acute coronary syndrome and acute myocarditis, facilitating risk stratification with myocardial tissue characterization [90,91]. In the context of acute CRS, one study demonstrated an association between microvascular myocardial injury assessed by contrast-enhanced CMR and increased risk of AKI in patients with ST-elevation myocardial infarction [92]. The value of CMR in the clinical management of acute CRS is yet to be unraveled by further studies.

### 4.2. Kidney Sonographic Biomarkers for Prognosis 

Renal resistive index and intrarenal venous flow pattern evaluated by Doppler imaging have demonstrated potential values in prognosis of acute CRS. Increased resistive index of the renal artery was found to be helpful in predicting AKI in patients after major cardiac surgery (type 1 CRS), and in patients with septic shock or in critical conditions (type 5 CRS) [93,94,95]. Since the key role of renal venous congestion has been recognized, intrarenal venous flow has attracted increasing interests [96,97,98]. The patterns of intrarenal venous flow were applied to identify renal hemodynamic disturbances in heart failure [99,100]. The discontinuous patterns of intrarenal venous flow were found to be associated with increased right atrial pressure and had independent prognostic values in patients with non-ischemic heart failure [100]. A case report observed the change of intrarenal venous flow from a monophasic to a biphasic pattern in parallel with improvement in symptoms and renal function [101]. Results of a recent clinical trial suggested that both renal arterial resistive index and intrarenal venous flow might offer guidance on the diagnosis and treatment of type 1 CRS [102].

### 4.3. Preclinical Kidney MRI Biomarkers of AKI

Multiparametric kidney MRI has been studied to characterize microstructural changes in AKI in recent years. Although the value of MRI biomarkers of AKI in the context of CRS remains to be investigated, there have been studies detecting the pathophysiological alterations in AKI. These techniques may facilitate early identification of AKI, which is one of the most challenging issues in clinical management of acute CRS. It has been well accepted that renal parenchymal hypoperfusion and hypoxia are closely associated with development of all forms of AKI [103]. BOLD technique by MRI has been used to evaluate intrarenal oxygenation in animal models and patients with AKI [104,105]. Renal hypoxia detected by BOLD MRI has been reported in contrast-induced AKI, renal allografts with acute tubular necrosis, sepsis-associated AKI, and other nephrotoxin-induced AKI [105]. Significantly lower perfusion of the renal cortex and medulla detected by ASL has been reported in AKI patents in comparison with healthy volunteers [106]. ASL was studied as an alternative to dynamic contrast-enhanced MRI for quantitative renal perfusion measurements in a rat model of AKI [107]. Moreover, the combination of BOLD and ASL techniques may help to achieve a better characterization of the primary cause of AKI, as the tissue oxygenation assessed by BOLD is significantly influenced by renal perfusion [108]. A study of 15 healthy volunteers demonstrated that ASL is capable of detecting renal hemodynamic change after a single-dose pharmacological intervention with captopril, highlighting the potential of ASL to provide mechanistic insights into the pharmacotherapy of kidney diseases [83]. DWI and T1 mapping techniques are potentially beneficial for the evaluation of AKI in acute CRS. Decreased ADC, alterations in IVIM parameters and diffusion anisotropy demonstrated by DTI have been shown in animal models of AKI [105]. Prolonged renal cortical T1 relaxation time and decreased corticomedullary difference was found in AKI and the cortical T1 values were positively correlated with stages of renal function [109].

## 5. Application of Imaging Biomarkers in Chronic CRS

### 5.1. Cardiac Imaging Biomarkers of CKD-Associated Cardiomyopathy

Echocardiography is currently recommended by the Kidney Disease Improving Global Outcomes (KDIGO) guidelines for all patients initiating dialysis, due to the high prevalence of underlying abnormalities among patients with CKD [110]. Characteristic cardiac changes in CKD include left ventricular (LV) hypertrophy, ventricular dilatation, cardiac dysfunction, and myocardial fibrosis [111]. However, TTE has disadvantages in identification and surveillance of LV myocardial mass and volumes in CKD. TTE tends to overestimate LV mass in comparison with CMR, and the wider intra- and inter-operator variability of TTE is disadvantageous for observation of subtle and gradual cardiac changes in CKD [112]. In addition, the impact of kidney transplantation on LV mass has been controversial, suggesting that the interventions to prevent type 4 CRS might need to be moved to earlier phase of CKD [113]. LV global longitudinal strain (GLS) is more sensitive than LV ejection fraction as a marker of subtle LV dysfunction [114,115,116], and is associated with an increased risk of mortality in predialysis and dialysis patients [117]. Previous studies demonstrated decreased LV-GLS and diastolic strain rates by TTE in CKD patients [114,118,119,120,121]. LV diastolic dysfunction can be diagnosed and graded by TTE, based on mitral valve annular e’ velocity, average E/e’ ratio, left atrium volume index, and peak tricuspid regurgitation velocity [122]. However, our recent study suggests that subclinical changes in myocardial tissue composition may exist even when no systolic or diastolic dysfunction was detected by TTE in patients on peritoneal dialysis [123].

CMR has the unique value of detecting myocardial fibrosis, which was found in more than 90% of patients with CKD in a postmortem study [124]. Increased myocardial native T1 value has been observed in patients with early phase CKD and in end-stage CKD patients when compared with healthy controls [125,126,127,128]. Two previous studies revealed higher myocardial T2 values in ESRD patients than those in healthy controls [123,129]. Decreased MR-derived LV global longitudinal strain and circumferential strain were also reported in patients with early CKD and in end-stage CKD patients [123,125,126,127,128,130]. Increased native T1 value has been found to be associated with LV global strain [123,125,126]. Most recently, a study of 134 pre-dialysis patients without diabetes or myocardial ischemia showed that native myocardial T1 values and serum biomarkers of myocardial fibrosis increase with advancing CKD stages, independent of left ventricular afterload [51]. These findings suggest that myocardial fibrosis might be a pharmacological target for the treatments in CKD patients, and might improve prognosis by mitigating the effects of CRS.

CAD and myocardial infarction with non-obstructive coronary artery can be involved in both type 2 and type 4 CRS. Coexistence of CAD and CKD and with comorbidities such as diabetes often manifests in these patients as ‘silent’ ischemic heart disease without typical anginal chest pain. Earlier CMR study with late gadolinium enhancement showed a mixed pattern of subendocardial infarction and diffuse fibrosis in patients with advanced CKD, reflecting the dual myocardial diseases [131]. Considering the increased risk of post-contrast acute kidney injury and nephrogenic systemic fibrosis in patients with severe renal dysfunction, non-contrast imaging techniques are preferred to identify CAD in CRS. The utility of echocardiography, nuclear cardiac imaging, CMR, CT, and hybrid imaging for diagnosis of CAD in patients with CKD has been thoroughly discussed in a most recent literature review [63]. 

### 5.2. Preclinical Kidney MRI Biomarkers of CKD with Potential Value in CRS

Kidney imaging has scarcely been studied in the context of chronic CRS, since cardiovascular abnormalities are more related to mortality. However, imaging biomarkers of CKD in general may have potential value in clinical management of chronic CRS, especially in early diagnosis and monitoring disease progression. 

Conventional kidney ultrasonography and MRI can hardly identify preclinical renal injury in chronic CRS. Although previous studies suggest that kidney size is associated with glomerular filtration and kidney function reserve [132], the relationship between kidney volume and function is not proportional, since the kidneys have a substantial functional reserve and homeostatic adaptive mechanisms [133]. Functional and tissue characterization MRI techniques may open new possibilities for future studies of chronic CRS. Feasibility of a multiparametric renal MRI protocol—including ASL, T1 mapping, DWI, and BOLD—for patients with CKD has been demonstrated [134]. There have been studies with histological evidences demonstrating that cortical ADC values measured by DWI correlated well with cortical fibrosis and chronic lesions [135,136,137,138]. Lower renal perfusion, significant higher cortical and medullary T1 value with reduced cortico-medullary differentiation have been observed in CKD patients compared with healthy volunteers [134,139]. The degree of cortical hypoxia indicated by decreased T2* value in BOLD was correlated with the extent of fibrosis on renal biopsy in one study [136]. However, another study failed to identify significant associations between T2* and eGFR or CKD stage in 342 patients with CKD [140]. A recent prospective study of 112 patients with CKD demonstrates that low cortical oxygenation indicated by BOLD-MRI is an independent predictor of renal function decline over the subsequent three years [141]. 

Type 5 chronic CRS secondary to diabetes are attracting increased attention these years, in which diabetic nephropathy has been of particular interest. Chronic hypoxia is one of the major contributors of parenchymal fibrosis and CKD in diabetes [142,143]. Lower renal ADC value and higher FA have been reported in early stage of type 2 diabetic nephropathy in comparison with healthy volunteers [144], and ADC value was correlated with urinary and serum biomarkers [145]. Decreased renal perfusion quantified by ASL was seen in patients with diabetes mellitus in comparison with healthy controls, despite normal eGFR and absence of overt albuminuria [146]. A multiparametric MRI study demonstrated significantly lower renal perfusion assessed by ASL in patients with diabetes and stage 3 CKD, and lower perfusion with lower response to furosemide in patients with progressive CKD [147]. 

## 6. Opportunities for Comprehensive Imaging Assessment of Heart and Kidneys in Future Studies

Ultrasonography remains the most versatile, accessible, and cost-effective modality for the assessment of CRS. MRI, on the other hand, is the most promising one-stop modality for the structural and functional evaluation of both heart and kidneys. Future studies aiming at finding novel biomarkers for CRS may incorporate serial ultrasonography or non-contrast MRI scans for simultaneous evaluation of heart and kidneys in their study design. 

In the context of acute CRS, a combination of TTE and renal sonography can be used to assess the heart and kidneys synchronously. The evaluation of right-sided congestion and intra-renal blood flow by Doppler imaging might offer incremental diagnostic and prognostic value together with circulatory and urinary biomarkers. Quantification of global ventricular strain may have the potential of early identification of cardiac dysfunction in type 3 CRS. 

The unique role of MRI in assessment of interstitial fibrosis in both the organs might complement the use of molecular biomarkers and provide new insights in the diagnosis and treatment of CRS in the future. For institutions with well-developed infrastructures for multiparametric MRI, a combined non-contrast protocol assessing the heart and kidneys in a single scan session could be considered in future studies for patients at risk of or with CRS. Myocardial T1 mapping and T2 mapping together with renal T1 mapping and DWI can provide information on the extent of fibrosis in heart and kidneys [148], which is postulated to be the key driver of chronic CRS. ASL and BOLD can reflect tissue perfusion and oxygenation in the kidneys, offering opportunities to detect preclinical hemodynamic alterations. Myocardial strain derived from CMR cine images can be used to identify early impairment of cardiac function in type 2 and type 4 CRS. With consistent scan parameters and the absence of ionizing radiation or contrast agents, non-contrast MRI is the ideal modality for longitudinal tracking of pathophysiological changes in CRS, as well as for monitoring of therapeutic response without excessive biopsies. 

## 7. Summary

Despite endeavors to improve clinical outcome over the past decade, hospitalization rate, symptom burden, and mortality in patients with dual burden of heart and kidney diseases are still high [8]. Meanwhile the practical need for better prevention and management of CRS is imminent. CRS is a growing health, economical and societal problem as the fast increasing number of aging population lead to higher prevalence of heart and kidney diseases. Due to the multiple interconnected pathophysiological mechanisms of CRS, it is conceivable that biomarkers or interventions targeting single mechanisms are inadequate. Multi-modality and multiparametric imaging techniques have been applied for cardiovascular diseases and kidney diseases and offer opportunities for the evaluation of CRS. A consecutive and synchronous imaging strategy tracing the natural history of CRS can be encouraging for future directions. We propose a multidisciplinary approach involving cardiologists, nephrologists, and radiologists to improve the prospect of research studies and clinical management of cardiorenal syndrome in the future. 

## Figures and Tables

**Table 1:** Classification of cardiorenal syndrome.

Classification	Timing	Descriptions	Examples
Type 1 (acute cardiorenal)	Acute	Heart failure causing AKI	Acute decompensated heart failure resulting in AKI, acute ischemic heart disease, valvulopathy or arrhythmia causing cardiogenic shock and AKI
Type 2 (chronic cardiorenal)	Chronic	Chronic heart disease causing CKD	Chronic heart failure causing CKD
Type 3 (acute renocardiac)	Acute	AKI leading to acute cardiac dysfunction	AKI due to glomerulonephritis or urinary tract obstruction causing acute heart failure, acute coronary syndrome or arrhythmia
Type 4 (chronic renocardiac)	Chronic	CKD leading to chronic cardiac abnormalities	CKD-associated cardiomyopathy
Type 5 (secondary)	Acute or Chronic	Systemic diseases causing acute or chronic dysfunction of heat and kidneys	AKI and acute heart failure induced by sepsis or critical conditions, CKD and cardiac abnormalities in diabetes mellitus, cirrhosis, amyloidosis, vasculitis, etc.

AKI, acute kidney injury; CKD, chronic kidney disease.

**Table 2:** Current difficulties in diagnosis and management of cardiorenal syndrome.

Main Challenges in All Types of CRS	
Early diagnosis and prognosis Preventing or reversing the adverse impacts of CRS Difficulties in distinguishing CRS from other cardiovascular and renal comorbidities	
**Specific Difficulties in Acute and Chronic CRS**	
**Acute CRS**	**Chronic CRS**
Current diagnostic criteria hinders early detection of AKI Difficult to differentiate between true kidney injury and pseudo-worsening of kidney function Lack of sensitive tools to assess treatment effects and to track the progression from AKI to CKD	Lack of overt symptoms of cardiovascular diseases in CKD Lack of sensitive tools to identify and monitor the progression of cardiovascular involvement when conventional assessments remain normal Standard treatment is less effective in reducing cardiovascular mortality in CKD patients than in the general population.

**Table 3:** Modalities and techniques for cardiovascular and renal imaging related to cardiorenal syndrome.

	Ultrasonography	Magnetic Resonance Imaging	Computed Tomography	Nuclear Imaging
**Assessment of Heart**				
Conventional	1. 2-dimentioanl measurement of cardiac chamber size, estimation of ventricular function 2. Valvular morphology and function, ventricular wall motion 3. Estimation of hemodynamic biomarkers by Doppler imaging	1. Gold-standard measurement of chamber size and volume, ventricular systolic function, myocardial mass by cine imaging 2. Moderate to severe valvular abnormalities 3. Quantification of myocardial perfusion with contrast agent and/or vasodilator 4. Quantification of myocardial fibrosis and infiltration by late gadolinium enhancement	1. Calculation of calcium score 2. Evaluation of coronary artery morphology by CT coronary angiography using contrast agent	1. SPECT myocardial perfusion imaging is the most commonly used tool to diagnose coronary artery disease in CKD 2. Absolute quantification of myocardial blood flow by PET 3. Coronary flow reserve and stress myocardial perfusion by PET
Advanced	1. 3-dimentional measurement of ventricular volumes and myocardial mass 2. Ventricular strain quantified by speckle-tracking 3. Improved structural and functional evaluation using ultrasonic enhancing agent	1. Non-contrast quantification of myocardial infiltration/deposition by T1 mapping and T2(*) mapping 2. Myocardial infiltration/deposition by extracellular volume fraction with contrast agent 3. Ventricular strain quantified by feature/tissue tracking 4. Non-contrast assessment of myocardial perfusion by dobutamine inotropic stress CMR, MR-compatible exercise stress CMR, myocardial ASL 4. Myocardial hypoxia by BOLD, diffusion by DWI, diffusion anisotropy by DTI	1. Functional imaging and myocardial perfusion using contrast agent 2. CT angiography-based fractional flow reserve of coronary arteries 3. Myocardial infiltration/deposition by extracellular volume fraction with contrast agent	1. PET quantitative analysis of myocardial glucose utilization 2. SPECT evaluation of myocardial fatty acid oxidation 3.Hybrid imaging such as SPECT-CT, PET-CT, PET-MRI can generate multiple biomarkers in one scan
**Assessment of kidneys**				
Conventional	1. Kidney length, estimated volume and echogenicity of cortex and medulla 2. Identify obstruction 3. Renal resistive index	1. Volumetric measurement 2. Depiction of renal cortex and medulla by conventional T1-weighted and T2-weighted imaging	1. Preferred for evaluation of kidney stones 2. Quantification of renal perfusion and GFR using contrast enhanced CT	1. Differential diagnosis of AKI (prerenal AKI or acute tubular necrosis or postrenal AKI) by renal scintigraphy 2. Measurement of GFR 3. Measurement of renal blood flow
Advanced	1. Intrarenal blood flow pattern 2.Renal venous blood flow, renal venous impedance index, renal venous discontinuity 3. Ultrasonic enhancing agent to assess renal perfusion	1. Parenchymal oxygenation by BOLD 2. Noncontrast renal perfusion by ASL 3. Microstructural changes evaluated by DWI, DTI and T1/T2 mapping 4. Quantification of renal perfusion and GFR using dynamic contrast enhancement	1. Dual energy CT for tissue characterization	1. Renal SPECT-CT for assessment of GFR 2. Renal PET with novel radiotracers for faster and more accurate quantification of GFR
Radiation	None	None	Yes	Yes
Contrast agent and safety	Mirobubbles to enhance ultrasound signals; safe	Gadolinium-based contrast agents, associated with nephrogenic systemic fibrosis, not applicable in patients with AKI and ESRD	Iodinated contrast agents, increase the risk of contrast-induced nephropathy in patients with renal dysfunction	Radionuclide labeled agents, safe.
Strengths in assessment of cardiorenal syndrome	1. Most versatile, accessible and cost effective modality to evaluate the heart and kidneys simultaneously. 2. Doppler imaging may generate hemodynamic biomarkers for diagnosis, prognosis and therapeutic evaluation, especially for acute CRS. 3. Suitable for serial imaging across the natural history of CRS	1. The most promising one-stop modality to evaluate structure, function and microstructural alterations in both heart and kidneys 2. Unique ability of quantitative assessment of fibrosis in both organs. Multiparametric scan to evaluate diffused infiltration/deposition, changes in perfusion, diffusion and oxygenation of heart and kidneys. 2. With consistent scan parameters and no radiation, non-contrast MRI is ideal for longitudinal tracking of cardiac and renal pathophysiological changes	Most widely used noninvasive technique for anatomical assessment of coronary artery disease	Important modality for evaluation of myocardial perfusion in coronary artery disease in patients with CKD, without the use of toxic contrast agent.
Limitations	Can be compromised by inadequate acoustic window, poor Doppler signals and operator-dependent variations	Expensive, prolonged acquisition time, requiring high compliance of patient, complicated post-processing procedures	Not suitable for longitudinal serial evaluation due to radiation, limited utility without contrast agent	Not suitable for longitudinal serial evaluation due to radiation, low spatial resolution, prolonged acquisition time, limited utility and accessibility

SPECT, Single-photon emission computed tomography; PET, positron emission tomography; CMR, cardiovascular magnetic resonance; CT, computed tomography; BOLD, blood oxygen level dependent; ASL, arterial spin labeling; DWI, diffusion weighted imaging; DTI, diffusion tensor imaging; AKI, acute kidney injury; CKD, chronic kidney disease; ESRD, end stage renal disease; GFR, glomerular filtration rate.
